# Gut microbiome and plasma lipidome analysis reveals a specific impact of *Clostridioides difficile* infection on intestinal bacterial communities and sterol metabolism

**DOI:** 10.1128/mbio.01347-24

**Published:** 2024-08-27

**Authors:** Ricardo Arcay, Maria Barceló-Nicolau, Loreto Suárez, Luisa Martín, Rebeca Reigada, Marcus Höring, Gerhard Liebisch, Carmen Garrido, Gabriel Cabot, Helem Vílchez, Sara Cortés-Lara, Elisa González de Herrero, Carla López-Causapé, Antonio Oliver, Gwendolyn Barceló-Coblijn, Ana Mena

**Affiliations:** 1 Microbiology Department, Hospital Universitari Son Espases, Palma, Balearic Islands, Spain; 2 Institut d’Investigació Sanitària Illes Balears (IdISBa, Health Research Institute of the Balearic Islands), Palma, Balearic Islands, Spain; 3 Research Unit, University Hospital Son Espases, Palma, Balearic Islands, Spain; 4 Internal Medicine Department, Hospital Universitari Son Espases, Palma, Balearic Islands, Spain; 5 Institute of Clinical Chemistry and Laboratory Medicine, University Hospital Regensburg, Regensburg, Bavaria, Germany; 6 Gastroenterology Department, Hospital Universitari Son Espases, Palma, Balearic Islands, Spain; Louis Stokes Veterans Affairs Medical Center, Cleveland, Ohio, USA

**Keywords:** *Clostridioides difficile*, recurrent CDI, gut microbiota, fecal microbiota transference, plasma lipidome, cholesterol metabolism

## Abstract

*Clostridioides difficile* infection (CDI) causes alterations in the intestinal microbiota, frequently associated with changes in the gut metabolism of bile acids and cholesterol. In addition to the impact on microbiome composition and given the metabolic changes occurring during CDI, our work focuses on the importance to know the effects at the local and systemic levels, both during the infection and its treatment, by paying particular attention to plasma lipid metabolism due to its relationship with CDI pathogenesis. Specific changes, characterized by a loss of microbial richness and diversity and related to a reduction in short-chain acid-producing bacteria and an increase in bile salt hydrolase-producing bacteria, were observed in the gut microbiota of CDI patients, especially in those suffering from recurrent CDI (RCDI). However, gut microbiota showed its ability to restore itself after treatment, resembling healthy individuals, in those patients treated by fecal microbiome transfer (FMT), in contrast with those treated with antibiotics, and displaying increased levels of *Eubacterium coprostanoligenes,* a cholesterol-reducing anaerobe. Interestingly, changes in plasma lipidome revealed a global depletion in circulating lipids in CDI, with the largest impact on cholesteryl esters. CDI patients also showed a specific and consistent decrease in the levels of lipid species containing linoleic acid—an essential fatty acid—which were only partially recovered after antibiotic treatment. Analysis of the plasma lipidome reflects CDI impact on the gut microbiota and its metabolism, evidencing changes in sterol and fatty acid metabolism that are possibly related to specific alterations observed in gut microbial communities of CDI patients.

**IMPORTANCE:**

There is increasing evidence about the influence the changes in microbiota and its metabolism has on numerous diseases and infections such as *Clostridioides difficile* infection (CDI). The knowledge of these changes at local and systemic levels can help us manage this infection to avoid recurrences and apply the best therapies, such as fecal microbiota transfer (FMT). This study shows a better restoration of the gut in FMT-treated patients than in antibiotic-treated patients, resembling healthy controls and showing increased levels of cholesterol-reducing bacteria. Furthermore, it evidences the CDI impact on plasma lipidome. We observed in CDI patients a global depletion in circulating lipids, particularly cholesteryl esters, and a specific decrease in linoleic acid-containing lipids, an essential fatty acid. Our observations could impact CDI management because the lipid content was only partially recovered after treatment, suggesting that continued nutritional support, aiming to restore healthy lipid levels, could be essential for a full recovery.

## INTRODUCTION

*Clostridioides difficile* infection (CDI) is the leading cause of nosocomial diarrhea and one of the main etiological agents of community-acquired diarrhea associated with antibiotics, ranking as one of the nosocomial pathogens with the greatest impact and the most difficult to control (1, 2). In this sense, the rapid spread of epidemic and sometimes hypervirulent strains is associated with greater severity and number of recurrences (3–6). Despite correct clinical and therapeutic management of patients, the risk of recurrence in CDI ranges from 20% after initial infection to 60% after multiple recurrences. These recurrences are progressively more frequent and serious, representing a marked decrease in the quality of life of patients as well as a significant increase in hospital stay with the consequent health cost this entails. The increasing presence of hypervirulent and antimicrobial-resistant strains favors the worrisome increase in CDI incidence and the number of patients with recurrent episodes, who are more challenging to manage and may require alternative therapies such as fecal microbiome transference (FMT).

Several studies describe how antibiotics alter the intestinal microbiota, leading to a loss of microbial diversity, some taxa, or metabolic shift, with a final reduced colonization resistance against different pathogens, including *C. difficile* (7, 8). In this sense, class-specific changes in microbial composition, emergence of drug-resistant bacteria, and effects on gut immunity have been described (9, 10). The antibiotic-induced reduction in the taxonomic and functional diversity provides optimum conditions for sporulation, spore germination, and toxin production of *C. difficile*. Hence, knowledge of the microbiome, populations affected, and the metabolism changes can improve the understanding of the mechanisms favoring *C. difficile* recurrences. Anaerobes seem to be relevant and important mediators of colonization resistance, and certain species—predominantly from the *Bacillota* and *Actinomycetota* phyla antagonize specific infections via direct and indirect mechanisms. Short-chain fatty acids (SCFA)—mainly represented by acetate, propionate, and butyrate—play a fundamental role in the regulation of host gene expression, inflammation, differentiation, and apoptosis (11, 12). Similarly, the expression of *C. difficile* toxins is regulated by many nutrients found in the gut, including butyrate (13). The normal microbiota is protective against gastrointestinal infections by invading pathogens and produces substances with antimicrobial properties, including SCFAs, secondary bile acids (BA), and bacteriocins (14). In agreement with its relevance, in the last years, the field has made a significant effort to understand the impact of CDI on microbiome metabolism. However, a less explored aspect is the impact at the systemic level, beyond the gastrointestinal tract. By searching for biomarkers that could convey systemic alterations, we focused on plasma composition, particularly its lipid composition.

Thus, the lipid profile of the colon epithelium and plasma of patients with colon cancer is very sensitive to the patient’s health status, being able, for example, to segregate patients with malignant polyps from those with benign polyps (15–17). Based on this, we hypothesized that the changes occurring in the gut microbiota during the infection course and its treatment could also be reflected in plasma lipid composition. This study analyzes and compares the intestinal microbiota and plasma lipidome of healthy individuals with patients at different stages of CDI infection, primary (PCDI) or recurrent (RCDI), and also with several types of treatments, including antimicrobial (AT) and FMT. Differences between lipid biomarkers and gut composition, depending on the stage of infection and treatment, may lead to a better understanding and management of CDI.

## RESULTS

### Demographic and clinical data

**Fig 1:**
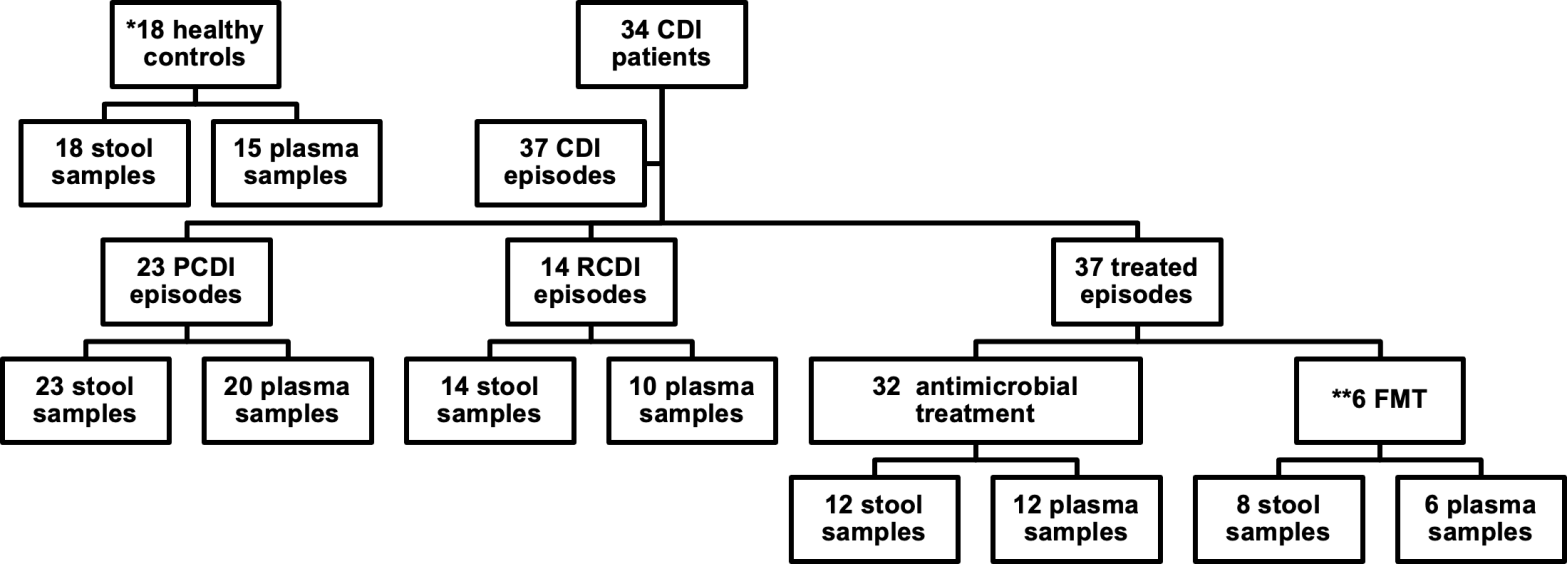
Study design. Total number of subjects included in the study, including recruited controls, CDI patients, and stool and plasma samples for the microbiome and lipidome analysis.

A total of 35 CDI patients and 20 healthy controls were considered and signed the informed consent during the studied period. Finally, 34 CDI patients and 18 healthy controls, including eight FMT donors, were included in the analysis. Those excluded were two healthy controls, one because of diabetes, and the other did not provide samples; and one CDI patient who started CDI treatment before obtaining samples. A total of 37 different CDI episodes, belonging to the 34 CDI patients, were included in the study since different episodes of the same patient were also considered when possible. Type of infection (primary or recurrent), treatment (AT or FMT), and the final number of stool and plasma samples included for the analysis are summarized in Fig. 1. All the most relevant data related to the studied subjects and their CDI episodes are summarized in Tables 1 and 2.

**TABLE 1:** Relevant demographic and clinical data of study subjects

Subjects		Patients (*n* = 34)	Healthy controls (*n* = 18)
Gender	Male	14 (41.1%)	9 (50%)
	Female	20 (58.8%)	9 (50%)
Age	Mean	67	47
	≥65 yrs	22 (64.7%)	4 (22%)
Health-care contact < 6 months		23 (67.6%)	0 (0%)
Immunocompromised condition^*a*^		22 (64.7%)	0 (0%)
Immunosuppressive treatments^*b*^		15 (44.1%)	0 (0%)
IBD^*c*^		7 (20.5%)	0 (0%)
Metabolic syndrome		15 (44.1%)	0 (0%)
PPI^*d*^		19 (55.8%)	0 (0%)

^*a*^ Due to the patient’s own condition.

^*b*^ Due to treatment administration.

^*c*^ IBD, inflammatory bowel disease.

^*d*^ PPI, proton pump inhibitor.

**TABLE 2:** Relevant data related to CDI episodes

CDI episodes		(*n* = 37)
Previous CDI episodes	<2	28 (75.6%)
	≥2	9 (24.3%)
Previous antibiotic treatment (<3 months)	No	10 (27%)
	Yes	27 (73%)
No. of previous antibiotic classes	1	9 (40.7%)
	2	7 (25.9%)
	≥3	11 (40.7%)
Antibiotic classes	Beta-lactams	20 (74%)
	Quinolones	12 (44.4%)
	Glycopeptides	5 (18.5%)
	Nitroimidazoles	4 (14.8%)
	Oxazolidinones	4 (14.8%)
	Macrolides	2 (7.4%)
	Lincosamides	2 (7.4%)
	Rifamycins	2 (7.4%)
	Fosfomycin	1 (3.7%)
	Aminoglycosides	1 (3.7%)
Stool EIA *C. difficile* toxin detection	Positive	19 (51.3%)
	Negative	18 (48.6%)
PCR-ribotype	R106	6 (16.2%)
	R078	3 (8.1%)
	R005	2 (5.4%)
	R014	2 (5.4%)
	R020	2 (5.4%)
	R081	2 (5.4%)
	Other	20 (54%)
CDI treatment	FMT	5 (13.5%)
	Antibiotic	32 (86.4%)
	Metronidazole	5 (43.7%)
	Vancomycin	3 (37.5%)
	Fidaxomycin	6 (18.7%)

### The gut microbial composition differs depending on the infection stage

**Fig 2:**
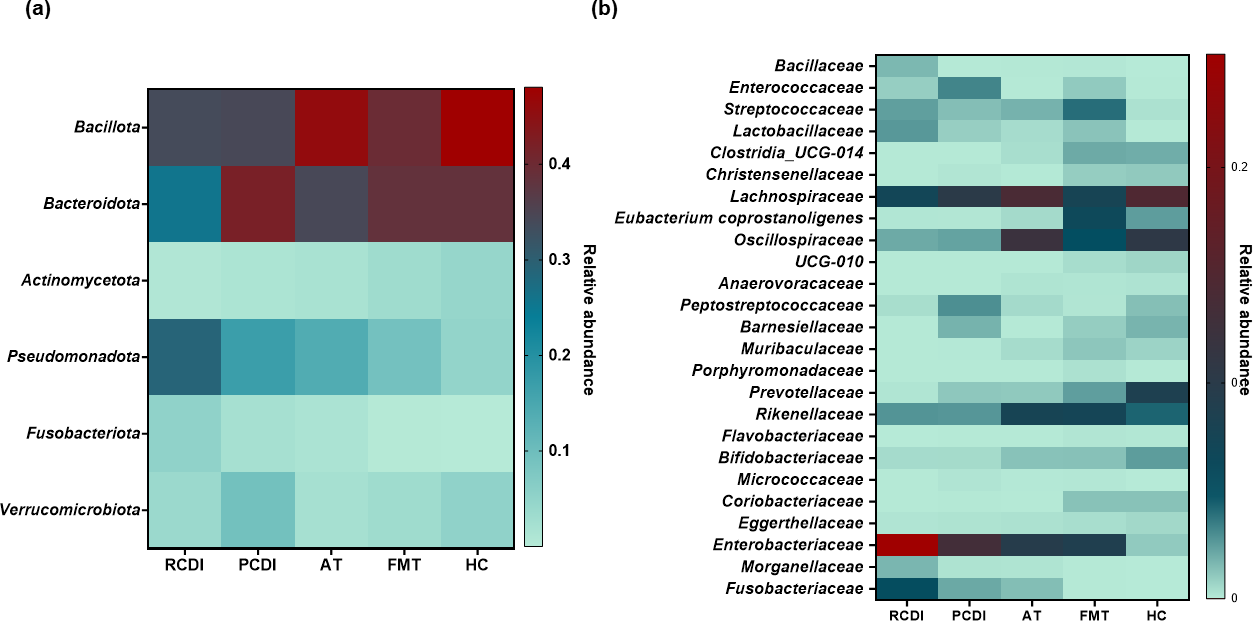
Heatmap representing observed differences at the phylum (a) and family (b) levels in relative abundance of the gut microbiota depending on the patient´s stage and type of CDI treatment (HC, healthy controls; CDI, *C. difficile* infection; AT, antimicrobial treatment; FMT, fecal microbiota transference therapy).

Differences in the relative abundance of relevant gut microbial classes were evidenced depending on the studied group of subjects. Overall, all the studied groups showed a reduction in the relative abundance of *Bacillota* and *Actinomycetota*, and an increase in *Pseudomonadota*, compared to healthy controls. The reduction of *Bacillota* in CDI patients was mainly associated with a decrease in relevant families of *Eubacteriales* (*P* < 0.05), while increased levels of *Pseudomonadota* were due to increased *Enterobacteriaceae* (*P* < 0.0001), and also *Morganellaceae* and *Fusobacteriaceae*, especially in RCDI (*P* ≤ 0.0002). Moreover, differences were observed between the different types of therapies since AT patients showed increased levels of *Enterobacteriaceae*, *Fusobacteriaceae*, or *Streptococcaceae* (*P* < 0.05)*,* that were not observed in FMT. Overall, a recovery of different *Clostridia*, *Bacteroidales*, *Coriobacteriaceae*, and *Morganellaceae* were observed in FMT-treated patients in contrast to AT patients where *Oscillospiraceae* was the only *Clostridia* family recovered. The main significant differences observed in the microbial relative abundance at different phylogenetic levels are represented in Fig. 2. Additionally, all the significant differences observed in the relative abundance for the different studied groups are summarized in Table S1.

**Fig 3:**
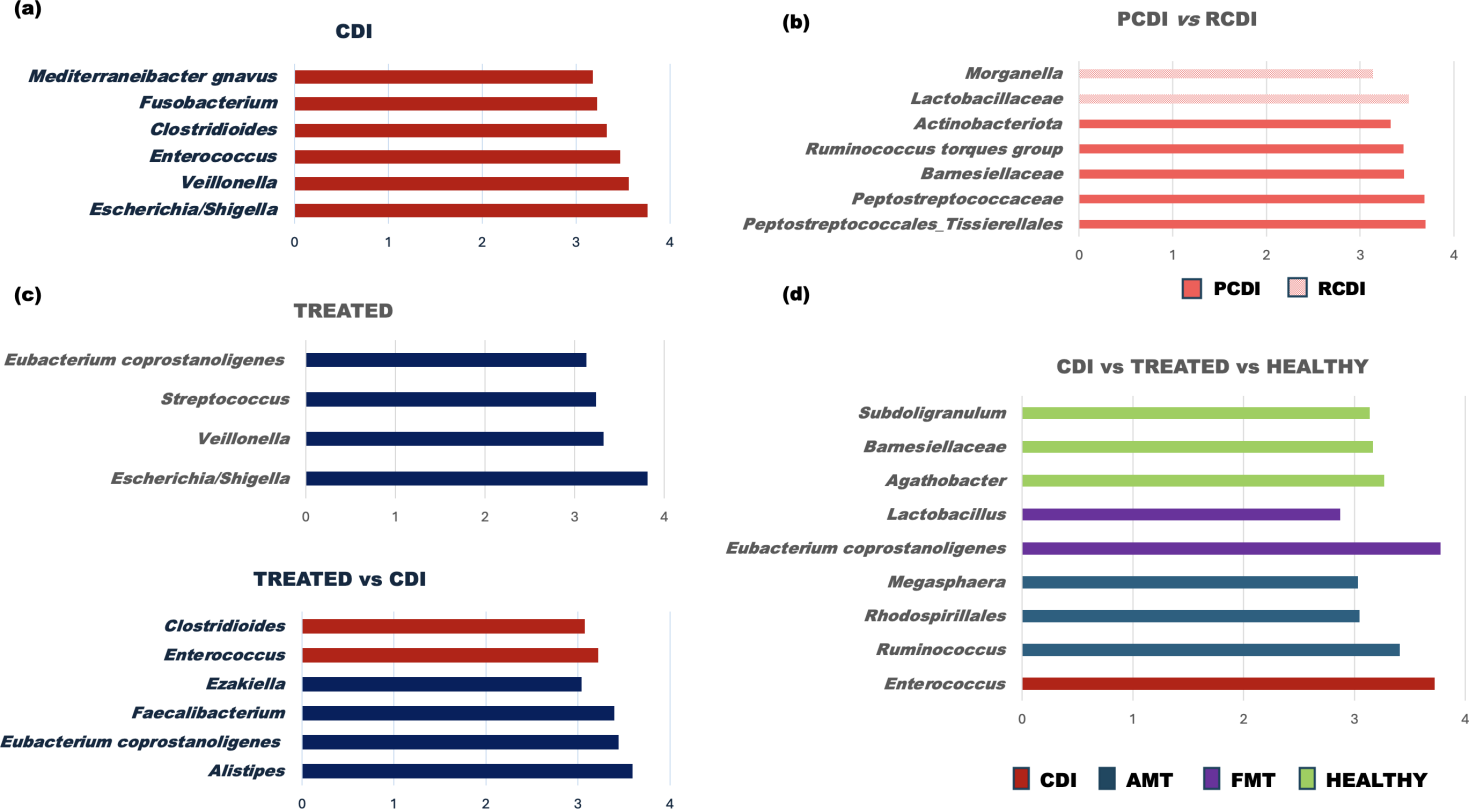
Differential abundance of the most significant gut microbial taxa depending on the patient´s stage performed by linear discriminant analysis effect size (LEfSe) and showing LDA scores. (a) Differential abundance in CDI patients compared to healthy controls (LDA > 3, *P* < 0.05); (b) differential abundance between primary (PCDI) and recurrent (RCDI) episodes (LDA > 3, *P* < 0.05); (c) differential abundance in treated patients regardless of type of treatment, compared to healthy controls and compared to *C. difficile*-infected patients (CDI) (LDA > 3, *P* < 0.05); (d) differential abundance of taxa comparing the different infection stages and therapies (LDA >2, *P* < 0.05).

Subsequently, the differential abundance was analyzed to find out markers of the clinical status (LDA > 3, *P* < 0.05). Noteworthy was the differential abundance of *Enterococcus* only in CDI patients, *Morganella* and *Lactobacillaceae* in recurrent episodes, or *Escherichia/Shigella* and *Veillonella* in CDI and treated patients. Treated patients showed differential abundance of the *Eubacterium coprostanoligenes* group, being especially notable in FMT-treated patients (Fig. 3).

### *C. difficile* alters gut microbiota, especially in recurrent infection

**Fig 4:**
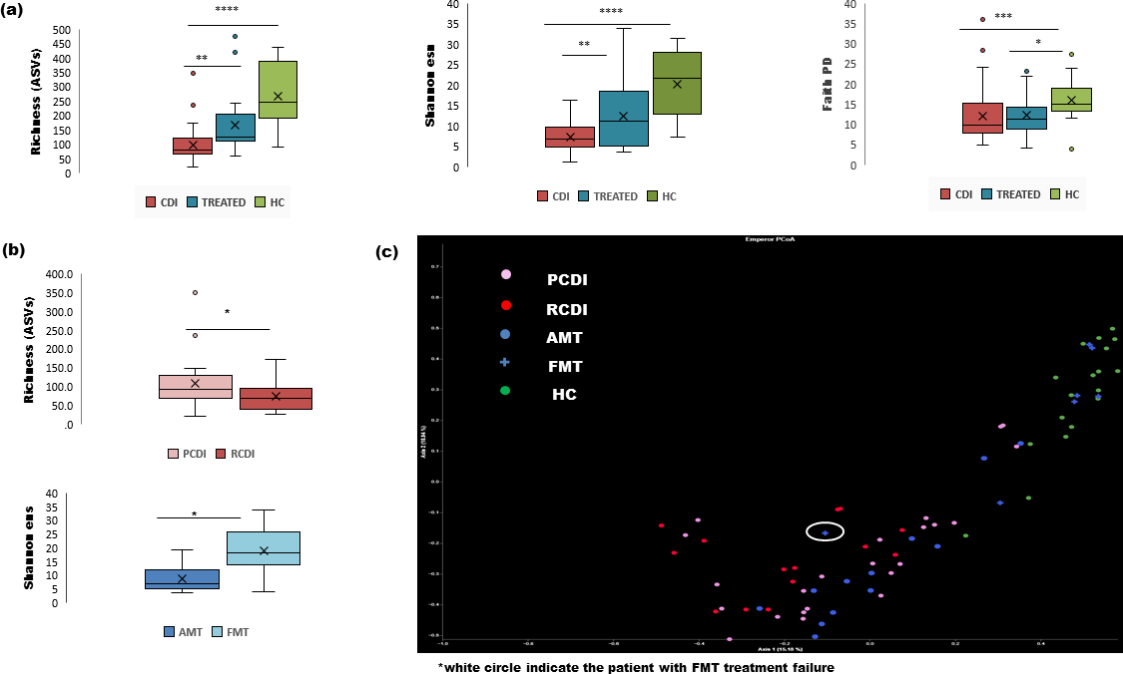
Alpha and beta diversity analysis of the different studied populations: (a) statistically significant differences were evidenced between healthy, CDI, and treated patients with reduced richness and diversity in CDI and reduced phylogenetic richness and microbial evenness in CDI and treated patients (*P* < 0.05 by Kruskal–Wallis); (b) statistically significant differences were also observed in phylogenetic richness (between patients with recurrent or primary CDI and between patients according to type of CDI treatment (richness, Faith, Shannon-ens, or Pielou index, *P* < 0.05 by Kruskal–Wallis); (c) beta diversity analysis also revealed significant quantitative dissimilarities between the different groups, including differences depending on the treatment (Bray–Curtis dissimilarity by PERMANOVA, *P* = 0.001), but also qualitative differences, except for FMT and HC (Jaccard distance by PERMANOVA, *P* = 0.001).

Alpha-diversity analysis showed clear differences among the groups for all the studied parameters (Table 3). Overall, reduced microbial richness, diversity, and microbial compositional evenness were evidenced in CDI patients compared to healthy controls and treated patients (*P* < 0.005) (Fig. 4a). Moreover, RCDI patients displayed more reduced phylogenetic richness (quantitatively and qualitatively) in their microbial communities than patients with primary episodes (measured by number of observed features, and Faith index) (Fig. 4b).

**TABLE 3:** Medians of alpha diversity-measured parameters for the different stages studied

Stage	Observed features	Shannon effective number	Faith PD	Pielou evenness
RCDI	69	6.98	8.55	0.59
PCDI	94	7.56	10.17	0.60
Treated	125	12.32	11.32	0.63
AT	119.5	7.03	10.62	0.59
FMT	214.5	18.12	14.13	0.67
HC	247	21.76	15.14	0.69

### FMT-treated patients show better microbiota restoration than patients treated with antibiotic therapy

Six CDI patients included in the analysis received FMT therapy. An immediate cure was observed in all patients, while only five achieved a definitive cure at 8 weeks. One of the patients suffered a new recurrent CDI episode after 20 days of FMT. There were no significant adverse effects, and no differences were observed between related and unrelated donors.

One of the most relevant differences observed in the alpha diversity analysis was the type of treatment, revealing lower phylogenetic diversity, richness, and microbial evenness in AT patients compared with FMT-treated patients. Interestingly, no significant differences were observed between FMT-treated patients and healthy controls (Fig. 4b).

Beta diversity analysis also revealed clear treatment-dependent dissimilarities, including quantitative differences (abundance) in microbial communities among all groups (*P* = 0.001). Furthermore, qualitative differences were evidenced (presence/absence) between all the studied groups except for FMT-treated patients and healthy controls (*P* = 0.001) (Fig. 4c).

It is noteworthy that the patient with FMT failure showed clear dissimilarities with the rest of the FMT-treated patients, and lower diversity and richness than the other FMT-treated patients (white circle in Fig. 4c). In fact, compositional analysis also evidenced differences in microbial relative abundances compared to the rest of FMT-treated patients and donors.

Interestingly, surveillance for multidrug-resistant microorganism (MDR) bacteria colonization status showed that two patients were colonized by MDR *Pseudomonas aeruginosa* and an ESBL *Klebsiella pneumoniae* before the FMT. However, follow up MDR surveillance after the FMT demonstrated the eradication of MDR bacteria in both cases.

### *C. difficile* infection affects plasma lipid composition

The development of high-throughput lipidomic analytical methods has prompted numerous studies aiming to identify clinical biomarkers. In this context, plasma lipidome is a rich source of biomarkers for diseases, including cardiovascular diseases (18), cancer (16, 19–23), and neurobiological disorders (24). Aiming to identify lipid profiles specifically associated with CDI, we analyzed plasma lipidome by flow injection analysis–mass spectrometry (FIA-MS). The analysis enabled the detection of more than 190 lipid species belonging to four main lipid categories, which in turn are subdivided into several lipid classes: glycerophospholipids, including phosphatidylcholine (PC), lysophosphatidylcholines (LPC), alkyl-phosphatidylcholines (PC O), phosphatidylethanolamines (PE), lysophosphatidylethanolamine LPE, alkyl-phosphatidylethanolamines (PE O), and phosphatidylinositol (PI); sphingolipids, including sphingomyelins (SM) and ceramides (Cer); glycerolipids, which include diacylglicerides (DG) and triacylglicerides (TG); and sterol lipids, in particular, free cholesterol and cholesteryl esters (CEs) (Fig. S1) (25). In global terms, in healthy controls, CEs were the most abundant lipid, accounting for 41.0% of total plasma lipids. Next in abundance were free cholesterol, PC, and TAG (16.7%, 15.8%, and 12.5%, respectively), and LPC and SM (6.1% and 4.8%), while the rest of the families accounted for 3.0% of the total lipids (see Table S2 for detailed data of all lipid classes).

**Fig 5:**
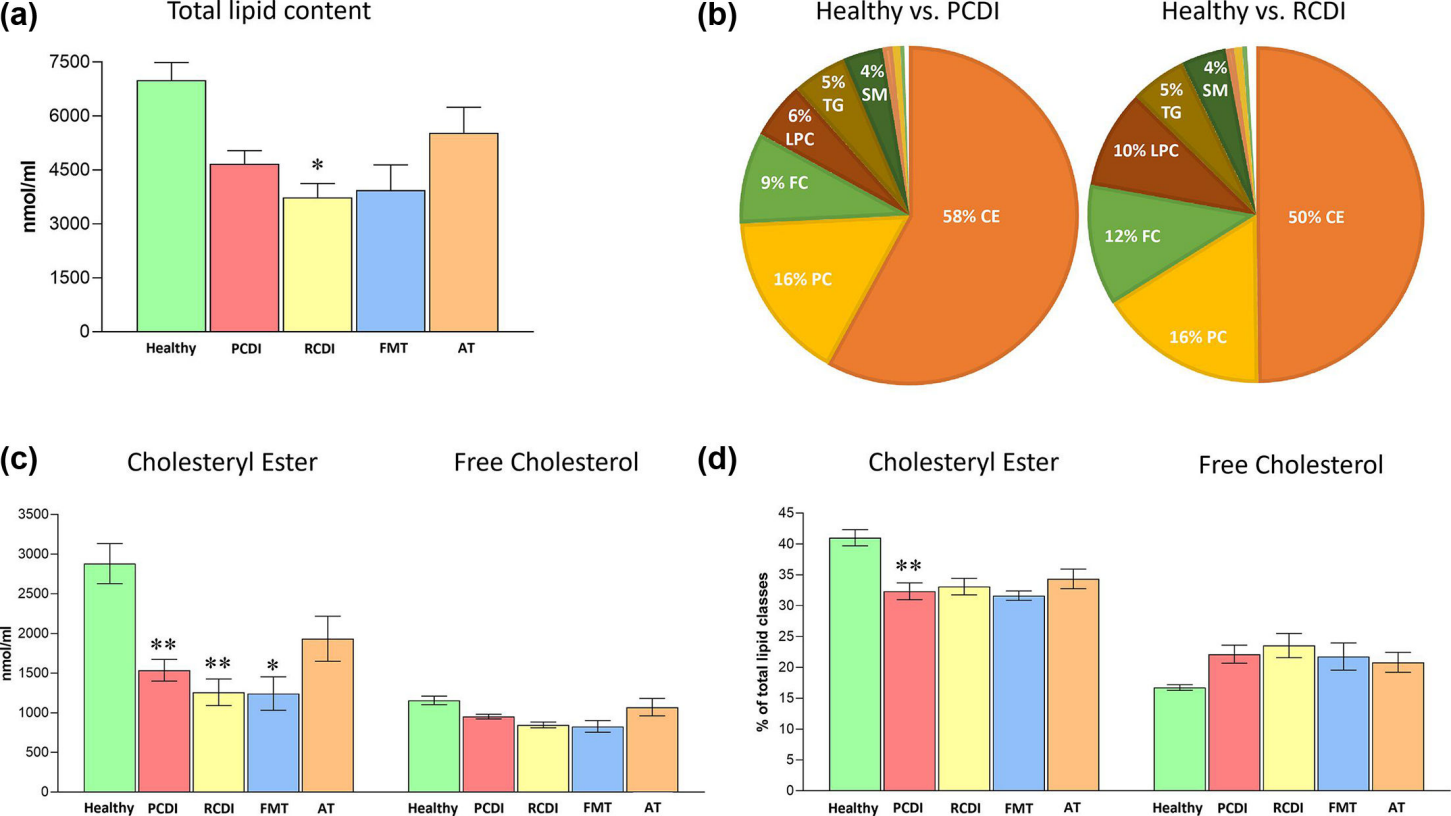
Specific impact of *C. difficile* infection on plasma total lipid levels. (a) Impact of the infection on total lipid content in plasma; (b) specific impact of CDI infection on sterol lipids, particularly on CE; (c, d) CE and FC content in plasma in mass or relative levels, respectively. Values are expressed as nmol/mL and percentage of total lipid classes (mean ± SEM), healthy, *n* = 6; PCDI, *n* = 18; RCDI *n* = 6; FMT *n* = 3; AT *n* = 11. Statistical significance was assessed using one-way ANOVA followed by Bonferroni post-test. The asterisk indicates a significant difference in the healthy control and the rest of the groups (PCDI, RCDI, and treated). *P < 0.05; **P < 0.01. Detailed results are included in Table S2. Abbreviations: CE, cholesteryl esters; FC, free cholesterol; LPC, lysophosphatidylcholine; PCDI, primary CDI; RCDI, recurrent CDI; SM, sphingomyelin; AT, antimicrobial treatment; FMT, fecal microbiota transference therapy; TG, triacylglycerides.

**Fig 6:**
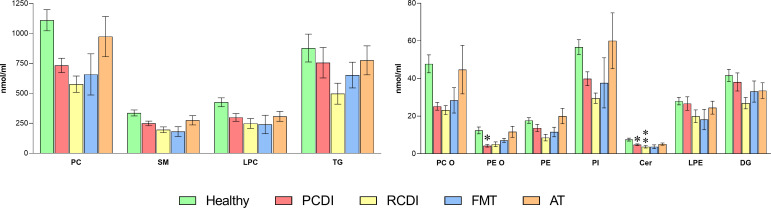
Impact of *C. difficile* infection on plasma phospholipid composition at the level of lipid class content. Values are expressed as nmol/mL and percentage of total lipid classes (mean ± SEM), healthy, *n* = 6; PCDI, *n* = 18; RCDI *n* = 6; FMT *n* = 3; AT *n* = 11. Statistical significance was assessed using one-way ANOVA followed by Bonferroni post-test. The asterisk indicates a significant difference between healthy control and the rest of the groups (PCDI, RCDI, and treated). *P < 0.05; **P < 0.01. Detailed results are included in Table S2. Abbreviations: Cer, ceramide; DG, diacylglycerol; LPC, lysophosphatidylcholine; LPE, lysophosphatidylethanolamine; PC, phosphatidylcholine; PCDI, primary CDI; PC O, alkylphosphatidylcholine; PI. phosphatidylinositol; PE, phosphatidylethanolamine; PE O, alkylphosphatidylethanolamine; RCDI, recurrent CDI; SM, sphingomyelin; AT, antimicrobial treatment; FMT, fecal microbiota transference therapy; TG, triacylglycerides.

**Fig 7:**
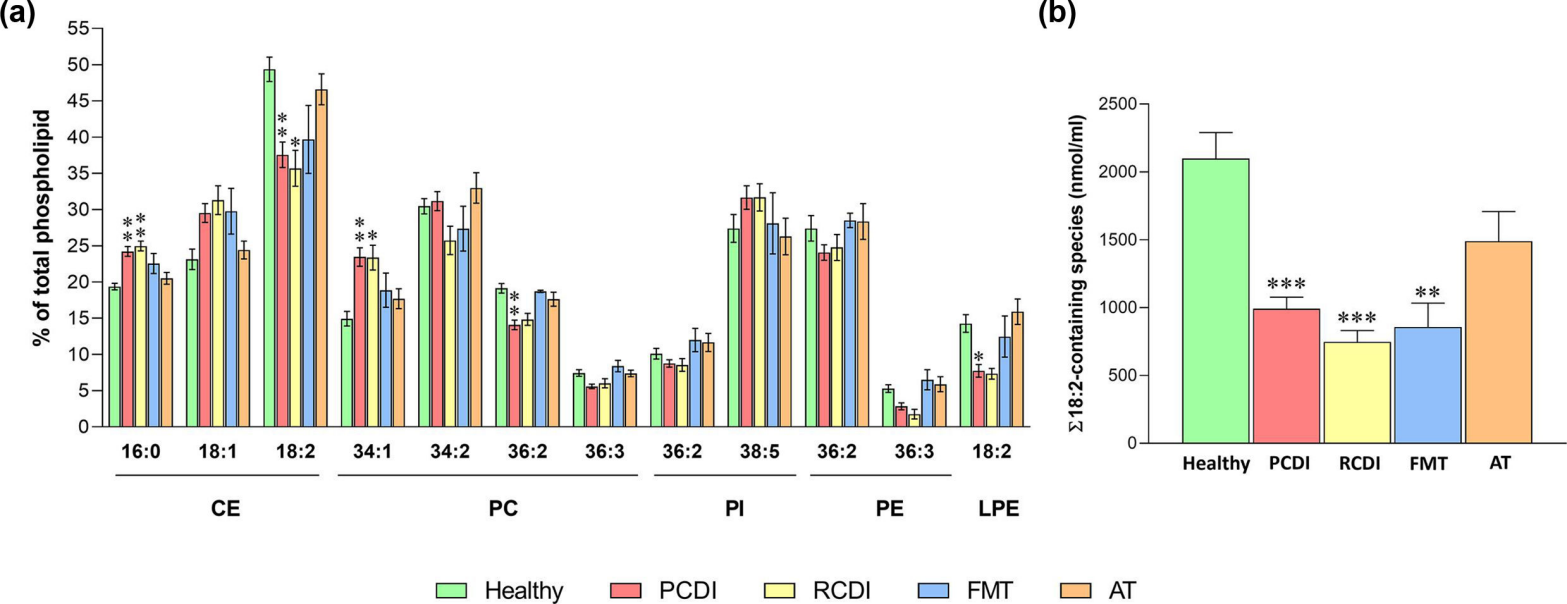
Specific impact of *C. difficile* infection on plasma phospholipid composition at the species level. (a) Summary of species following the same pattern of changes throughout several lipid classes: decrease in 18:2-containing species and increase in SFA and MUFA-containing species. Values are expressed as a percentage within each class (mean ± SEM), healthy, *n* = 6; PCDI, *n* = 18; RCDI *n* = 6; FMT *n* = 3; AT *n* = 11. (b) Impact of the infection on plasma 18:2-containing lipids. Values are expressed as nmol/mL (mean ± SEM), healthy *n* = 6; PCDI, *n* = 18; RCDI *n* = 6; FMT *n* = 3; AT *n* = 11. Statistical significance was assessed using one-way ANOVA followed by Bonferroni post-test. The asterisk indicates a significant difference between healthy control and the rest of the groups (PCDI, RCDI, FMT and AT). *P < 0.05; ** *P* < 0.01; ***P < 0.001. Detailed results are included in Table S2. Abbreviations: CE, cholesteryl esters; LPE, lysophosphatidylethanolamine; PC, phosphatidylcholine; PCDI, primary CDI; PI, phosphatidylinositol; PE, phosphatidylethanolamine; RCDI, recurrent CDI; AT, antimicrobial treatment; FMT, fecal microbiota transference therapy.

**Fig 8:**
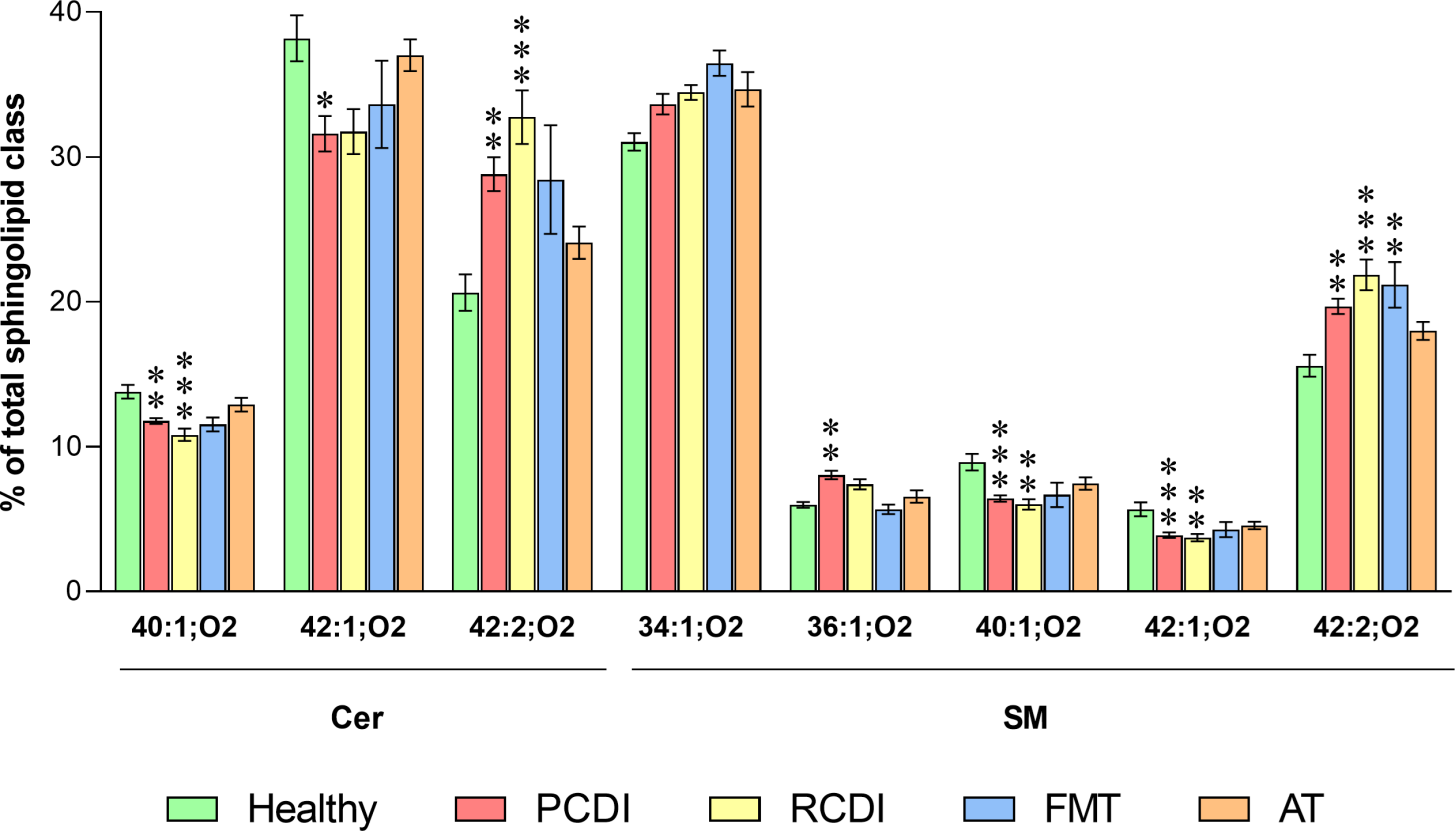
Impact of *C. difficile* infection on sphingolipid composition. Values are expressed as a percentage of the sphingolipid class (mean ± SEM), healthy, *n* = 6; PCDI, *n* = 18; RCDI *n* = 6; FMT *n* = 3; AT *n* = 11. Statistical significance was assessed using one-way ANOVA followed by Bonferroni post-test. The asterisk indicates a significant difference between healthy control and the rest of the groups (PCDI, RCDI, FMT, and AT). *P < 0.05; **P < 0.01; ***P < 0.001. Detailed results are included in Table S2. Abbreviations: AT, antimicrobial treatment; Cer, ceramide; FMT, fecal microbiota transference therapy; PCDI, primary CDI; RCDI, recurrent CDI; SM, sphingomyelin.

**Fig 9:**
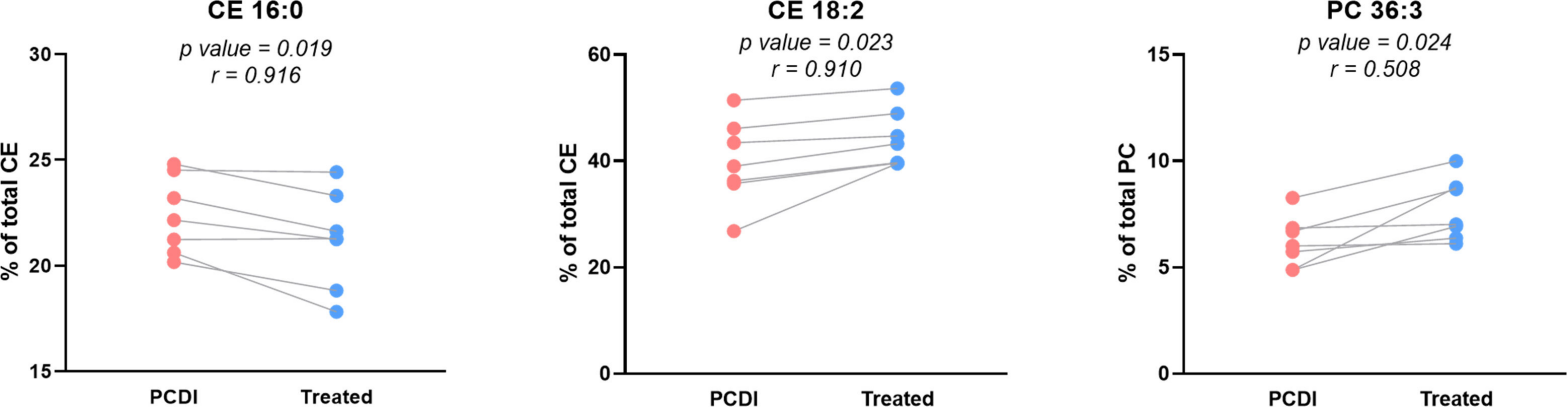
Comparison of paired infected and treated plasma lipidome from PCDI patients. Plots showing relative abundance of selected lipid species in paired CDI patients. *P* values (paired *t*-test) are shown for each comparison; *n* = 7. In this case, all patients were treated with antimicrobial therapy. Abbreviations: CE, cholesteryl esters; PCDI, primary CDI.

The analysis revealed a profound impact of CDI on the plasma lipidome, drastically affecting the total amount of circulating lipids and relative composition (Fig. 5 to 9; Table S2). Compared to healthy controls, the total lipid content decreased from 6,997 to 4,676 and 3,739 nmol/mL in PCDI and RCDI, implying a decrease of 33.2% and 46.6%, respectively (Table S2; Fig. 5a). Unexpectedly, total lipid levels were not recovered in FMT-treated patients, while in AT-treated patients, they tended to recover. The lipid depletion was consistent with the malfunctioning of the small and large bowels in the infected patients, which could lead to inefficient lipid absorption. Importantly to this study, 50% to 58% of this decrease was due to the specific impact on CE content (Fig. 5b through d).

Although quantitatively, a decrease occurred throughout all the lipid classes, the relative composition showed that CE and free cholesterol levels (accounting for approximately 60% of total circulating lipids) were differently altered in infected patients (Table S2). Thus, while CE decreased by 5%–6% in the affected groups, FC levels increased by a similar percentage. Altogether, these results reinforce the specific impact of *C. difficile* infection on sterol metabolism, specifically affecting CE (Fig. 5d). We also observed a significant reduction in the mass of PE O (66.2% and 59.4% in CDI and RCDI groups, respectively) and Cer (35.9 and 50.8 %) (Fig. 6). Further, although not statistically significant, the rest of the classes also showed a solid tendency to decrease in CDI patients, with the most noticeable changes found in PE O (66.2%), PC O (47.3%), PC (34.0%), LPC (29.8%), PI (29.7%), SM (26.0%), and PE (22.5%). After treatment, the patients showed a general tendency to increase plasma lipid content, and in AT-treated patients, some reached control levels (Fig. 6).

Next, we investigated the CDI impact at the lipid species level (Table S2). The quantitative analysis revealed a global impact on all lipid species in both PCDI and RCDI groups. Conversely, the levels of almost all glycerophospholipid species were re-established in AT-treated patients, whereas this recovery was not observed in neutral lipid species (Table S2).

Although in terms of mass, all lipid species decreased in patients infected by *C. difficile*, the results expressed as a percentage revealed a common pattern (Fig. 7a). Thus, in infected patients, species containing linoleic acid (18:2n-6) were consistently decreased in CE (CE 18:2) and throughout the glycerophospholipid family (PE 36:2, PE 36:3, PC 36:2, PC 36:3, PC 34:2, PI 36:2, LPE 18:2), while their levels tended to recover only in AT patients. Remarkably, linoleic acid is an essential fatty acid that must be acquired by dietary means. Hence, the specific impact on 18:2n-6-containing species (Fig. 7b) would be consistent with impaired functioning of the bowels, where lipid absorption and stool passage occur.

The impact of the infection on the sphingolipid family followed a common profile (Fig. 8). While in terms of mass, all sphingolipid species were decreased in infected groups, the levels were recovered in AT-treated patients (Table S2). The composition described in relative terms showed that some Cer and SM species were affected following a similar pattern: while 34:1;O2 and 42:2;O2 increased, 40:1;O2 and 42:1;O2 decreased in infected patients.

In summary, our analysis clearly demonstrates a drastic impact on plasma lipid composition, with its content decreasing by 30%–50% in mass. Although all lipid classes were affected, the decrease in the CE pool was rather remarkable, linking to a severe alteration of the cholesterol metabolism at the systemic level. Similarly, a specific impact was observed on 18:2n-6-containing glycerophospholipids.

Finally, because of the solid differences found in plasma lipid composition, we evaluated inter-individual variation between PCDI and after treatment. Although our data pointed to significant lipid alterations, additional factors, such as age, diet, gender, and several comorbidities, may influence the observed changes. To assess this possibility, our data were analyzed by comparing paired PCDI and AT patients (*n* = 7), allowing us to evaluate inter-individual variation with greater precision. Consistent with the impact of CDI on CE, we identified two species, CE 16:0 and CE 18:2, showing a high correlation (*r* ≥ 0.7) between groups (Fig. 9), indicating that infection has an independent effect on the CE composition. We also analyzed some parameters related to the microbiome, although we did not find statistical differences (Fig. S2**,** first row).

## DISCUSSION

Although the evidence of compositional and metabolic changes occurring at the gut level during CDI is accumulating, there is still a need to delve into the association of these changes with *C. difficile* pathogenesis and the effects they may entail at the local and systemic levels. In line with several studies, our results support a loss in gut microbial richness, diversity, and evenness in CDI patients, especially in patients suffering from recurrent episodes (26–28). Differential abundance of gut microbial composition was found between primary and recurrent infections in our cohort of patients. These compositional differences could help to better understand why some patients suffer from a higher number of recurrences. In this context, some studies already describe how temporal gut microbial changes (29) or a small set of gut metabolites (30) enable recurrences in CDI patients to be predicted.

Differences in the gut microbial community composition between healthy individuals and CDI patients have already been described in the literature (28, 31, 32). These compositional alterations are mainly represented by a decrease in butyrate-producing groups (*Oscillospiraceae* and *Lachnospiraceae*), as well as *Clostridia* clusters IV, XIVa, or *Bacteroides*. Moreover, increased levels of *Enterococcus*, *Veillonella*, *Lactobacillus,* or bacteria of the *Gammaproteobacteria* class have also been reported in CDI patients (27, 31). In line with these studies, a decrease in several relevant families of *Eubacteriales,* related to beneficial functions, as butyrate production, like *Eubacterium*, or *Oscillospiraceae*, which are also related to propionate production, was observed in our cohort of infected patients (33). Concurrently, the increased *Pseudomonadota* observed in our CDI patients was significantly represented by *Escherichia/Shigella;* however, the increased levels in *Morganellaceae* in patients with recurrences should be highlighted as an important marker in these patients.

Our results also showed the microbial compositional restauration in treated patients, especially in those treated by FMT. Interestingly, microbiome analysis evidenced a better restoration of the gut microbiota in FMT than in AT patients. These patients showed no differences in alpha and beta diversity analysis from healthy controls, in contrast with the AT-treated patients. Moreover, all the patients treated by FMT, except one, achieved a definitive cure with no significant adverse events, and even MDR decolonization was evidenced in two patients. A differential abundance of some butyrate-producing bacteria, such as *Eubacterium coprostanoligenes* and *Ruminococcus* was observed in treated patients, with the differential abundance of *Eubacterium coprostanoligenes* specifically related to FMT-treated patients in contrast to CDI patients, where the presence of this group was practically non-existent. Of particular relevance is the fact that *Eubacterium coprostanoligenes*, a small, anaerobic, Gram-positive coccobacillus, has been described as a reducer of cholesterol to coprostanol (34). Some other phylotypes that have been correlated with this important metabolite are members of the *Lachnospiraceae* and *Oscillospiraceae* families, which are reduced in CDI patients but partially restored in treated patients. The involvement of cholesterol and bile acid metabolism in the resistance mechanisms to *C. difficile* colonization has already been highlighted in numerous works (35). In this sense, there are different data demonstrating that entry of *C. difficile* to colonocytes is cholesterol dependent (36), that toxin A binding to target cells is facilitated by cholesterol-enriched lipid rafts (37), or that a number of sterols and bile acids can inhibit *C. difficile* binding (38, 39). Although the precise mechanisms underlying colonization resistance remain unknown, it is thought that coprostanol may enhance resistance to CDI by decreasing the availability of cholesterol substrates for primary bile acid generation (40), and an inverse relationship between serum cholesterol levels and the coprostanol/fecal (41), knowledge of coprostanol-associated bacteria can be of particular interest for CDI patients. Cholesterol reduction by microbiota can be achieved by bile-salt hydrolases (42, 43). Moreover, FMT success has been previously correlated with an increase in bile-salt hydrolase copy number compared to levels prior to transplant, suggesting microbial modifications of primary BA (44).

It is worth stressing that some of the differentially abundant bacteria observed in CDI, such as *Enterococcus*, *Clostridioides, Ruminococcus gnavus* group, *Veillonella*, and *Fusobacterium*, are bile-salt hydrolase-producing bacteria involved in primary BA deconjugation. Especially notable are the increased levels of *Enterococcus*, which were even higher in recurrent infections, since it has been identified as one of the main genera of gut microbiota with bile-salt hydrolase activity (45). Moreover, a recent study describes how microbial interaction with *Enterococci* enhances the fitness of *C. difficile* by supplying fermentable amino acids, including leucine and ornithine, as well as its virulence by depletion of arginine (46).

BA are a major cholesterol-derived metabolite, with a plethora of functions, acting as signaling molecules regulating systemic endocrine functions including triglyceride, cholesterol, and possibly glucose homeostasis (47, 48). Consistent with this role, we observed a profound impact of *C. difficile* infection on the plasma lipidome, deeply affecting cholesterol metabolism and fatty acid profile. Thus, we demonstrated a drop in all circulating lipids (30%–50% in mass), i.e., glycerophospholipids, glycerolipids, and sterols, although CE was the most affected lipid class, both quantitatively and qualitatively (Fig. 5). Furthermore, in addition to having fewer circulating lipids, the fatty acid composition was selectively impoverished in linoleic acid (18:2n-6), an essential fatty acid and one of the most abundant in plasma (Fig. 7). To the best of our knowledge, this is the first time this effect has been described.

These observations agree with the impact that *C. difficile* infection has on one of the most important functions of the intestines—nutrient absorption—as infected patients commonly suffer from severe diarrhea and alterations in the intestinal barrier integrity. Intriguingly, while the plasma of AT-treated patients showed a strong tendency to recover the membrane lipid profile, the one of FMT patients remained altered. The results in plasma lipidome were very forceful, especially taking into account that the CDI patients have very different comorbidities, which in turn may have a different impact on systemic lipid metabolism. In addition, in our cohort, 62.2% were patients admitted to the hospital, while 37.8% were patients who did not acquire the infection in health facilities. Impressively, despite the heterogeneity among patients, the decrease in lipid contents was very consistent. These results also raise concerns regarding the functional state of the intestinal barrier, which seems that it could not be fully restored, and a closer follow-up of the nutritional status of the patient could be highly recommendable.

At the systemic level, CEs are the preferred form for the transport of plasma cholesterol and essential to avoid its toxic accumulation. In cells, two isoforms of sterol O-acyltransferase (SOAT1 and 2, previously named ACAT1 and ACAT2) account for CE synthesis. SOAT1 is present in several tissues, especially macrophages and adrenal and sebaceous glands, and is responsible for CE synthesis in arterial foam cells in human atherosclerotic lesions. SOAT2, exclusively expressed in hepatocytes and enterocytes (49), is involved in the CE supply to nascent lipoproteins. Conversely, cholesterol esterification in plasma occurs by a very different route. In high-density lipoproteins, CEs are synthesized by the transfer of fatty acids to cholesterol from position sn-2 of PC catalyzed by the enzyme lecithin:cholesterol acyl transferase (LCAT). Interestingly, a recent clinical study investigating the origin of CE in carriers of two mutant LCAT alleles, established that the lack of LCAT activity leads to CE poor in 18:2n-6, similar to that observed herein (50). Therefore, the impaired absorption of dietary fatty acids associated with CDI may interfere with adequate LCAT functioning by drastically diminishing substrate availability. In this scenario, SOAT2, known to synthesize CE enriched in oleic (18:1) and palmitic acid (16:0) (51), could become a major plasma CE contributor, giving rise to the observed profile fatty acid (Fig. 7; Table S2).

Our work is not exempt from some limitations as its single-center design makes it necessary for our results to be validated by other multicenter studies, including more patients, and achieving a complete follow-up from the beginning of the infection, recurrences, and treatment for the same patient in all cases. Even so, it was possible to carry out the follow-up for intestinal microbiota analysis in 17 infected patients and 10 patients for lipidome analysis. On the other hand, patients and controls were not matched according to age, gender, or other variables that could be relevant such as diet, comorbidities, medication, etc. However, it should be noted that the paired analysis in the follow-up of seven patients showed changes in the composition of the CE that could be attributed to the infection. Also, the mean age of the controls is somewhat younger than that of the patients, although we did not observe significant differences between age groups. Moreover, there are several other factors, such as diet, sports, medication, etc., as well as pathologies, such as inflammatory bowel disease (IBD), that can affect the microbiome or lipidome and should be considered confounding factors. It should be noted that none of the IBD patients in our cohort suffered severe episodes of their disease at the time of the study; hence, in all cases, diarrhea in those patients was treated as a cause of the CDI.

In conclusion, our study evidences alterations in the gut microbiota of CDI patients, mainly represented by the loss of microbial richness, diversity, and evenness, as well as a different class of SCFA-producing bacteria accompanied by an increase in some other bacteria, such as enterococci, which may play a relevant role in *C. difficile* pathogenicity. Interestingly, the results show that the changes observed in the gut microbiome are reflected by the plasma lipidome, since a specific decrease in cholesterol ester content and composition was observed in CDI patients. Beyond the mechanisms by which these changes are related, the restoration of some plasma compounds in treated patients could serve as a marker of infection status. Moreover, our results also evidence major microbial alterations depending on some conditions, such as suffering more than one recurrence, a fact that may have to be considered to apply appropriate treatments to reduce the effects on the intestinal microbiota. In this sense, our results also evidence better restoration of the gut microbiota in FMT-treated patients than in those treated with CDI antimicrobial therapies. Mechanisms associated with the greater effectiveness of this therapy still need to be completely deciphered, although our results reveal an association with the cholesterol-reducing bacterium, *Eubacterium coprostanoligenes*.

## MATERIALS AND METHODS

### Study cohort and sample collection

A cross-sectional case-control study was designed and conducted at the reference Hospital of the Balearic Islands (Spain), a 700-bed tertiary-care university hospital servicing approximately 750,000 inhabitants. Healthy volunteers not admitted or attended in the hospital and stool donors selected to perform FMT were considered for the control group. Cases were considered for in- and outpatients with CDI diagnosis, including primary or recurrent episodes. CDI diagnosis was performed following both clinical and laboratory criteria as previously described (52). Patients with colectomy or with a definitive ileostomy were directly excluded. Recurrent CDI episodes were considered as previously described (53).

Patients and controls were enrolled in the study between February 2018 and December 2020. Stool and plasma samples were obtained and frozen at −80°C for subsequent microbiota and lipidome analysis. Samples were classified depending on the moment of collection: (i) PCDI, (ii) RCDI, or (iii) treated, by AT or FMT. Therapy with FMT was considered only in patients after the second recurrence (at least a third CDI episode). Subsequently, treatment with oral vancomycin was started and withdrawn 48 h before the FMT. In those patients, stool samples for the microbiome analysis were collected and frozen just before the FMT procedure. Both related and unrelated donors were used, and transference was performed with fresh or previously frozen stools through colonoscopy, as described (54). Patients were followed up after 15 days to establish an “immediate cure” and over 8 weeks to define a “definitive cure.” During follow-up, samples were collected once the treatment was completed, when possible, at different times, ranging from 15 to 540 days from the beginning of treatment. In compliance with our protocol, surveillance for MDR colonization was performed in FMT-treated patients before and after transference by rectal swabs culture on MacConkey agar supplemented with 1 µg/mL of cefotaxime plus 50 µg/mL of vancomycin and 1 µg/mL of meropenem plus 50 µg/mL of vancomycin.

### Demographic and clinical data collection

Demographic and clinical data of the studied subjects and their CDI episodes were recorded together with other variables that were considered relevant for the microbiota and lipidome analysis (Tables 1 and 2).

### Gut microbiota analysis

Total DNA was extracted from fecal samples using the Qiagen Fast QiaAmp DNA Stool Mini Kit (Qiagen, Valencia, CA, USA) following the manufacturer’s protocol. Obtained DNA was then used for amplification of the V3 and V4 variable regions of the 16S rRNA gene according to the protocol previously described by Klindworth et al. (55). Libraries were sequenced on a MiSeq Illumina sequencer resulting in 2 × 300-bp paired-end reads (16S Metagenomic Sequencing Library Preparation protocol #15044223 Rev. B, available at https://support.illumina.com). The ZymoBIOMICS Microbial Community DNA Standard (D6305, Zymo Research) was included as a positive control. Raw sequenced reads were processed using the QIIME2v.2019.7 pipeline (56, 57). The DADA2 software, implemented in QIIME2, was used to remove noisy sequences, chimeras, singletons, and phiX reads with a quality score ≥20 from paired-end data. Taxonomic affiliations of Amplicon Sequence Variants (ASVs) were assigned using the naive Bayesian classifier integrated into the QIIME2 pipeline. The SILVA release 138 database clustered at 99% was used for taxonomic assignation (57). Alpha-diversity was estimated by Shannon effective number, microbial Evenness, Faith’s phylogenetic diversity, and observed features, within the QIIME2 software. Beta diversity metrics used were the Bray–Curtis and Jaccard distance. Nonparametric tests (Kruskal–Wallis and PERMANOVA) were applied to compare demographic and clinical data with the microbiota composition. To assess differential taxa as a potential biomarker of the patient state, an LEfSe analysis (linear discriminant analysis effect size) was performed. Except as stated otherwise in the Results section, all experiments in this study were run with LEfSe’s, a parameter for pairwise tests set to 0.05 for both class normality and subclass tests, and the threshold on the logarithmic score of LDA analysis was set to 2, although those with a score above 3 were considered of special interest (58).

### Lipidome analysis of plasma

All the samples used for lipid analysis were collected at least 1 month after the treatment was completed. Lipid extraction was performed according to the method of Bligh and Dyer in the presence of not naturally occurring lipid species as internal standards (59): PC 14:0/14:0, PC 22:0/22:0, PE 14:0/14:0, PE 20:0/20:0 (di-phytanoyl), PS 14:0/14:0, PS 20:0/20:0 (di-phytanoyl), PI 17:0/17:0, LPC 13:0, LPC 19:0, LPE 13:0, SM 18:1;O2/12:0, Cer 18:1;O2/14:0, Cer 18:1;O2/17:0, glucosylceramide (GlcCer) 18:1;O2/12:0, GlcCer 18:1;O2/17:0, D7-free cholesterol, CE 17:0, CE 22:0, TG 51:0, TG 57:0, DG 28:0, and DG 40:0. Chloroform phase was recovered by a pipetting robot (Tecan Genesis RSP 150) and vacuum dried. The residues were dissolved in chloroform/methanol/2-propanol (1:2:4 [vol/vol/vol]) with 7.5 mM ammonium formate.

Analysis of lipids was performed by direct FIA using a hybrid quadrupole-Orbitrap mass spectrometer (FIA-FTMS; high mass resolution) (60). TG, DG, and CE were recorded in positive ion mode, while multiplexed acquisition was used for the [M +NH_4_]^+^ of free cholesterol and D_7_-cholesterol (61). The mass range of negative ion mode was split into two parts: *m/z* range 400–650 to analyzed LPC and LPE and *m/z* range 520–960 for PC, PE, PI, SM, and Cer. Data processing details are described in Höring et al. (60) using the ALEX software (62), which includes peak assignment and intensity picking. Lipid species were annotated according to Liebisch et al. (63).

### Statistical analysis

Statistical comparison between the pathological condition groups and the healthy group was performed using one-way ANOVA followed by Bonferroni’s multiple comparisons test for the lipid analyses. Dunn’s multiple comparison Kruskal–Wallis tests were performed to compare the relative abundance between the different study groups for all taxonomic levels considered of interest. Results were plotted using Prism 8 (GraphPad Software Inc., USA).

## Data Availability

The data sets analyzed during the current study are available in the ENA repository, project PRJEB64491.

## SUPPLEMENTAL MATERIAL

Supplemental Figures mbio.01347-24-s0001.docx Fig. S1 and S2.

Table S1 mbio.01347-24-s0002.xlsx Significant differences in the relative abundance for the different studied groups.

Table S2 mbio.01347-24-s0003.xlsx Plasma lipid profile.

ASM does not own the copyrights to Supplemental Material that may be linked to, or accessed through, an article. The authors have granted ASM a non-exclusive, world-wide license to publish the Supplemental Material files. Please contact the corresponding author directly for reuse.
