# Supplementary material for: Gut microbiome and plasma lipidome analysis reveals a specific impact of Clostridioides difficile infection on intestinal bacterial communities and sterol metabolism
Source: mBio. 2024 Aug 27;15(10):e01347-24. doi: 10.1128/mbio.01347-24 (PMC11481895; doi:10.1128/mbio.01347-24)
Supplement: Supplemental Figures — Fig. S1 and S2. [file mbio.01347-24-s0001.docx]

Gut microbiome and plasma lipidome analysis reveals a specific impact of *Clostridioides difficile* infection on intestinal bacterial communities and sterol metabolism

Ricardo Arcay^a,c^, María Barceló-Nicolau^b,c^, Loreto Suárez^a,c^, Luisa Martín^c,d^, Rebeca Reigada^b,c^, Marcus Höring^e^, Gerhard Liebisch^e^, Carmen Garrido^f^, Gabriel Cabot^b,c^, Helem Vílchez^c,d^, Sara Cortés-Lara^b,c^, Elisa González de Herrero^a,c^, Carla López-Causapé^a,b,c^, Antonio Oliver^a,b,c^, Gwendolyn Barceló-Coblijn^b,c,#^ and Ana Mena^a,b,#^.

^a^Microbiology Department, *Hospital Universitari* Son Espases

^b^Research Unit, University Hospital Son Espases, Ctra. Valldemossa 79, E-07120 Palma, Balearic Islands, Spain

^c^*Institut d’Investigació Sanitària Illes Balears* (IdISBa, Health Research Institute of the Balearic Islands), Palma, Balearic Islands, Spain

^d^Internal Medicine Department, *Hospital Universitari* Son Espases, Palma, Balearic Islands, Spain

^e^Institute of Clinical Chemistry and Laboratory Medicine, University Hospital Regensburg, Regensburg, Germany

^f^Gastroenterology Department, *Hospital Universitari* Son Espases, Palma, Balearic Islands, Spain

Running Head: *C. difficile* alters microbiome and plasma lipidome

#Address correspondence to Ana Mena, ana.mena@ssib.es and Gwendolyn Barceló-Coblijn, gwendolyn.barcelo@ssib.es

SUPPLEMENTARY FIGURE 1


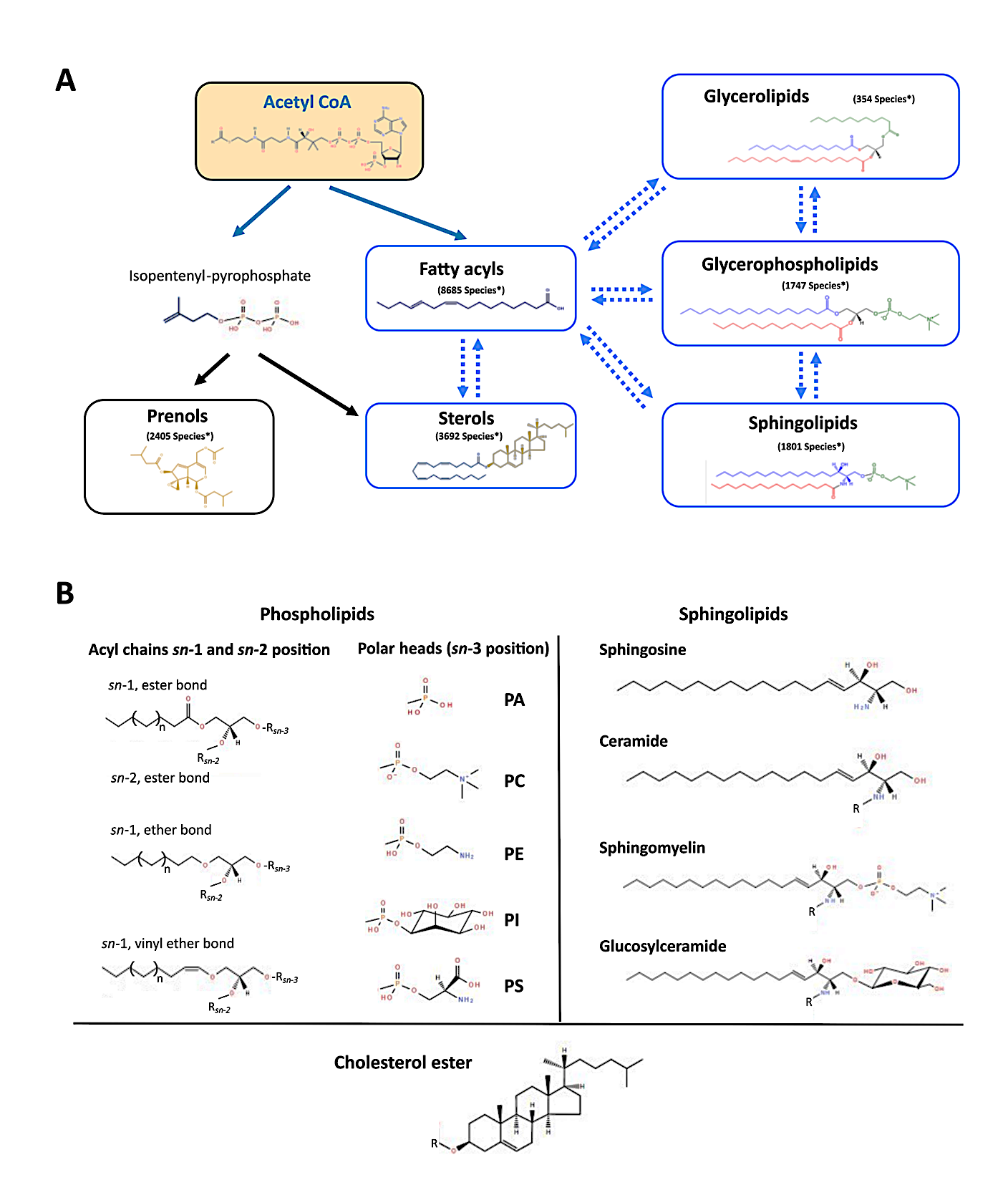


**Fig. S1. Lipids classification and structural characteristics.** A) Mammalian lipid categories. Metabolic interactions between the relevant lipid categories in mammals (in white), including the number of lipid species defined so far in each class. Comprehensive classification including the following eight categories: fatty acids, glycerophospholipids (or phospholipids), sterols, sphingolipids, glycerolipids, prenol lipids, saccharolipids, and polyketides [1]. These categories derive from a common precursor, acetyl-CoA (orange) [2]. (Lipid Maps Structure Database®, May 2023). B) Structural elements of glycerophosphate- and sphingoid-based lipids. Phospholipids: On the left, the different types of linkage established at sn-1 position between the fatty acid and the glycerophosphate backbone are shown. On the right, the most common polar heads (sn-3 position) found in mammalian cell membrane: phosphatidic acid (PA), phosphatidylcholine (PC), phosphatidylethanolamine (PE), phosphatidylserine (PS), and phosphatidylinositol (PI). Other less abundant or organelle specific lipid classes are phosphatidylglycerol (PG), cardiolipin (CL; exclusive of mitochondria), and bis(monoacylglycerol)phosphate (BMP; enriched in late endosomes). On the left, and “R sn-1 and sn-2” refers fatty acid moiety; on the right, “R sn-3” refers to glycerophosphate moiety. Sphingolipids: The most frequent sphingoid base is sphingosine, although there are also other structures such as sphinganines and 4-hydroxysphinganines. Depending on the polar head attached to the ceramide, different molecules are generated, such as sphingomyelins (phosphocholine), cerebrosides (glucose, galactose…), and gangliosides (oligosaccharides or sialic acid). In terms of the fatty acyl chain (R), in sphingolipids it is highly common to find saturated or monounsaturated fatty acids, which often contain a hydroxyl group at C2 position. “R” refers fatty acid moiety. Cholesterol: the principal sterol in all higher animals, serves as a precursor for the biosynthesis of steroid hormones, bile acid and vitamin D. Cholesterol can be esterified to a fatty acid moiety (R), generating cholesterol ester. Adapted from Bestard-Escalas et al. 2019 [2].

1. Fahy E, Subramaniam S, Brown HA, Glass CK, Merrill AH, Murphy RC, et al. A comprehensive classification system for lipids. J Lipid Res. 2005;46:839–62.

2. Bestard-Escalas J, Maimó-Barceló A, Pérez-Romero K, Lopez DH, Barceló-Coblijn G. Ins and Outs of Interpreting Lipidomic Results. J Mol Biol. 2019;431:5039–62.

SUPPLEMENTARY FIGURE 2


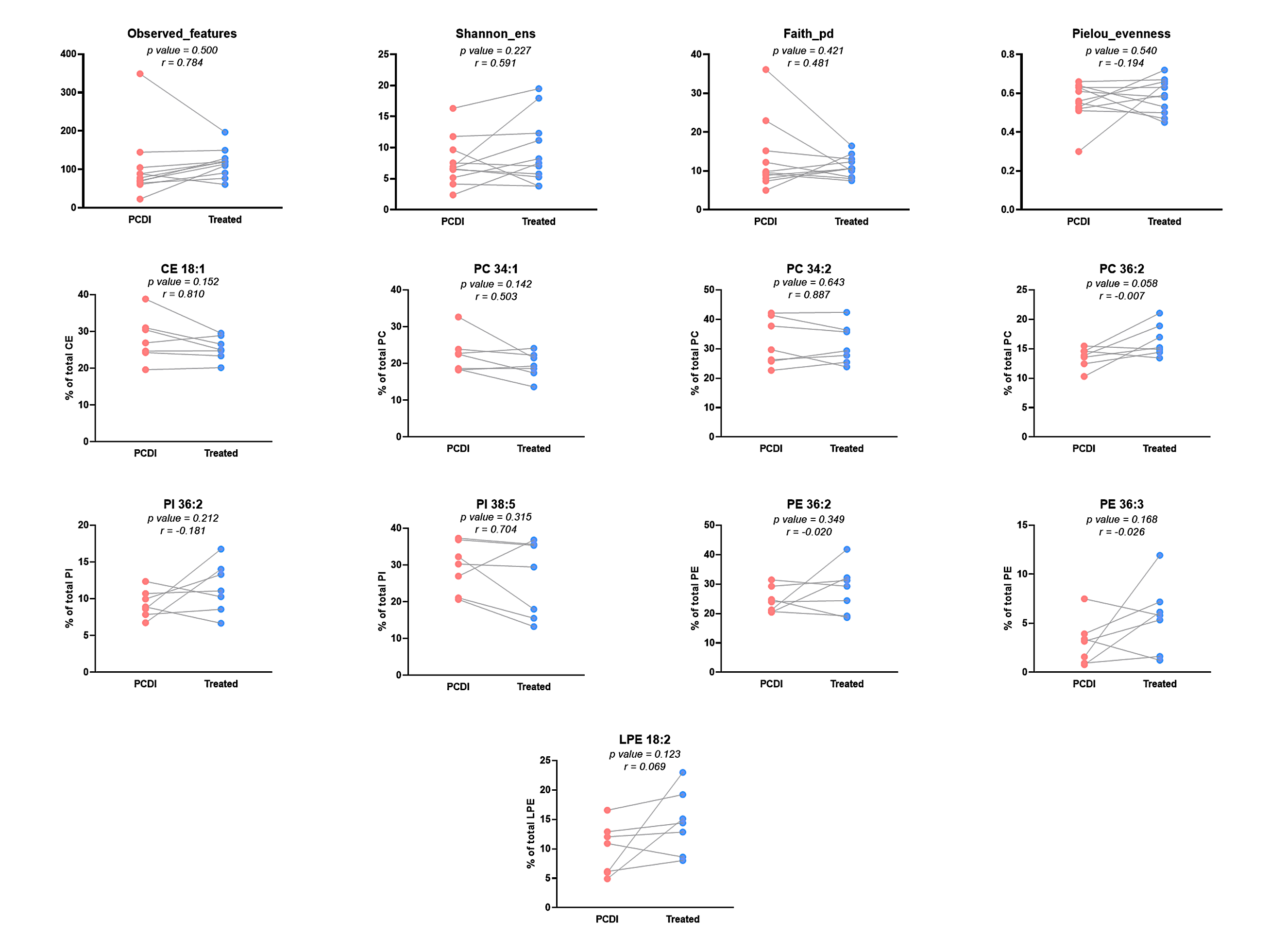
**Fig. S2. Comparison of paired infected and treated alpha-diversity microbiome parameters and plasma lipidome from CDI patients.** Plots showing relative abundance of selected lipid species in paired CDI patients. p values (paired t-test) are shown for each comparison, n=7. In this case all patients were treated with antimicrobial therapy. Abbreviations: CE: cholesteryl esters; LPE: lysophosphatidylethanolamine; PCDI: primary CDI; PC: phosphatidylcholine; PE: phosphatidylethanolamine; PI: phosphatidylinositol.
